# The Right Frontopolar Cortex Is Involved in Visual-Spatial Prospective Memory

**DOI:** 10.1371/journal.pone.0056039

**Published:** 2013-02-13

**Authors:** Alberto Costa, Massimiliano Oliveri, Francesco Barban, Sonia Bonnì, Giacomo Koch, Carlo Caltagirone, Giovanni A. Carlesimo

**Affiliations:** 1 Clinical and Behavioural Neurology Laboratory, Istituto Di Ricovero e Cura a Carattere Scientifico Santa Lucia Foundation, Rome, Italy; 2 Psychology Department, Palermo University, Palermo, Italy; 3 Clinical Neurology, “Tor Vergata” University, Rome, Italy; University College London, United Kingdom

## Abstract

The involvement of frontopolar cortex in mediating prospective memory processes has been evidenced by various studies, mainly by means of neuroimaging techniques. Recently, one transcranial magnetic stimulation study documented that transient inhibition of left Brodmann Area (BA) 10 impaired verbal prospective memory. This result raises the issue of whether the BA 10 involvement in prospective memory functioning may be modulated by the physical characteristics of the stimuli used. The present study aimed to investigate the role of the frontopolar cortex in visual-spatial PM by means of the application of inhibitory theta-burst stimulation. Twelve volunteers were evaluated after inhibitory theta-burst stimulation over left BA 10, right BA10 and CZ (control condition). In the prospective memory procedure, sequences of four spatial positions (black squares) each were presented. During the inter-sequence delay, subjects had to reproduce the sequence in the observed order (ongoing task forward) or the reverse order (backward). At the occurrence of a target position, subjects had to press a key on the keyboard (prospective memory score). Recall and recognition of the target positions were also tested. We found that prospective memory accuracy was lower after theta-burst stimulation over right BA10 than CZ (p<0.01), whereas it was comparable in left BA10 and CZ conditions. No significant difference was found among the three conditions on recall and recognition of target positions and on ongoing task performance. Our findings provide a novel strong evidence for a specific involvement of right frontopolar cortex in visual-spatial prospective memory. In the context of previous data providing evidence for left BA 10 involvement in verbal prospective memory, our results also suggest material-specific lateralization of prospective memory processes in BA 10.

## Introduction

In recent years, reliable data has been reported on involvement of the rostral frontal brain regions in mediating the ability to carry out delayed intentions (i.e., prospective memory; PM). Indeed, since Burgess et al. [1] and Okuda et al [2] published pivotal data on the effect of prefrontal cortex lesions on PM functioning and the functional brain correlates of PM, the role of the frontal pole in PM processes has been investigated and confirmed by means of functional neuroimaging techniques. In healthy individuals, metabolic and hemodynamic changes in Brodmann Area (BA) 10 were repeatedly found to be significantly related to PM task performance and the hypothesis of a dynamic interplay between the lateral and medial portions of this area was advanced (for a review of main studies, see [3]). In fact, the PM-related activity of the rostral frontal regions has been consistently detected in both subtraction and conjunction designs, regardless of the material adopted (e.g., shapes, words, figures) and the attentional demands of the procedures [2], [4]–[7]. Moreover, as Reynolds et al.’s data indicate, an increase in neural activity in this area may be specifically related to the implementation of PM processes and not to other cognitive processes (e.g., working memory) implicated in the execution of a PM task [8]. A clear-cut association between BA 10 activity and PM functioning was further confirmed in one transcranial magnetic stimulation (TMS) study. In this study the transient inhibition of left lateral BA 10 activity by means of off-line application of continuous Theta Burst Stimulation (cTBS) was shown to significantly worsen healthy subjects’ ability to activate prospective intention without affecting episodic recollection of the events [9].

To summarize, various evidence suggests that BA 10 activity has a key role in supporting PM abilities. However, it is less clear whether, within the frontal pole, we can hypothesize an hemispheric specialization on control of PM processes. Indeed, an asymmetrical pattern of neural activity related to the performance of a PM task, characterized by stronger activation of the left than the right frontal pole, has been evidenced by functional neuroimaging data [5], [6], [10], [11]. Some of these findings suggest basic dominance of the left hemisphere in PM operations regardless of the kind of material used (e.g., pictures, complex scenes or letter processing) [5], [12], [13]. Other studies have demonstrated different involvement of the left and right frontal pole depending on the integrational complexity and processing demands of the task, respectively [14], [15]. Nevertheless, some investigators failed to reveal significant hemispheric lateralization in BA 10 [4], [7].

Furthermore, as above noted, one previous study document that cTBS inhibition of activity of the left but not the right rostral frontal cortex significantly affected subjects’ ability to perform a verbal PM task [9]. On one side, this finding can be interpreted within the above views emphasizing the major involvement of the left rostral prefrontal cortex in PM processes [5], [13]. On the other side, as, in the paradigm used in that study subjects were required to elaborate words, it could be argued that finding was expression of the relative sensitivity of the activity of the frontopolar neurons to the physical features of the stimuli used. In this perspective, clarifying whether or not differential involvement of the left and right frontopolar cortices in PM performance is related to the verbal or visuospatial modality of stimuli presentation could provide valuable information to help clarify the neural correlates of PM and to elucidate BA 10 functioning. In fact, it has been hypothesized that involvement of BA 10 in cognitive tasks is substantially content-independent because it is related to the maintenance of abstract representations of complex rules [16]. This hypothesis is coherent with the idea of a hierarchical rostral-caudal organization of the prefrontal cortex in which the most anterior portions are involved in highly integrative, associative and supervisory operations [17]–[20]. Nevertheless, some recent findings are not in line with this hypothesis because they found that the neural activity in the anterior prefrontal cortex can be modulated by content-related manipulations [13], [14].

The present study aimed to investigate the role of the frontopolar cortex in visual-spatial PM by applying a cTBS paradigm. For this purpose, in one experiment with healthy individuals we evaluated the effect of trains of inhibitory cTBS [21] delivered over left and right BA 10 on PM performance. The general characteristics of cTBS and PM task procedures were the same as those used in the previously mentioned study [9]. In this case, however, in the PM task participants pressed a key at the appearance of a target position on the screen (instead of a target word) while they were engaged in a visual-spatial span task (ongoing activity). According to evidence supporting greater involvement of the left lateral frontopolar cortex in PM operations, we predicted that inhibitory cTBS delivered over left BA 10 would affect PM performance more than cTBS application over right BA 10. But, if a material-specific lateralization of PM processes exists in BA 10, the inverse pattern of effects should be observed.

## Materials and Methods

### Ethics Statement

All subjects gave their written informed consent to participate in the study. The experimental procedures were approved by the ethics committee of Foundation Santa Lucia.

### Subjects

Twelve healthy right-handed college students (6 women; mean age 21.2±2.5 years) took part in this experiment. All subjects gave their informed consent to participate in the study. All data were obtained in compliance with the regulations of our institution.

### TMS Procedure

**Figure 1:**
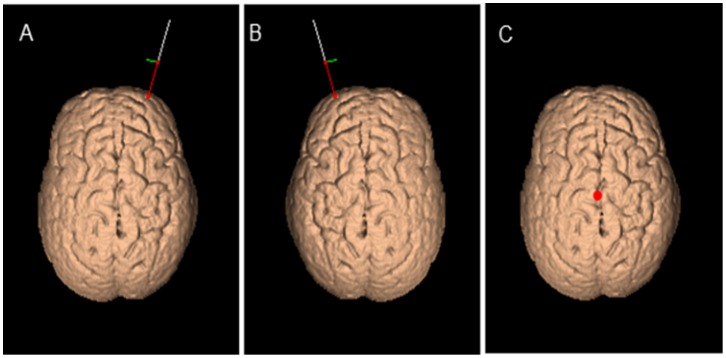
Coil positions for cTBS stimulation. The coil positions for Right BA 10 (MNI coordinates: x = 30.3±3.6; y = 50.9±3.8; z = 13.6±2.7; A) Left BA 10 (MNI coordinates: x = –30.3±3.4; y = 50.9±2.8; z = 13.6±1.9; B) and Cz (MNI coordinates: x = 0±3.8; y = 0±2.7; z = 94±4.1; C) are here illustrated. The sites of stimulation were defined by using Softaxic neuronavigation system.

We adopted a TMS procedure [22] described elsewhere (Figure 1; [9]). A MagStim Super Rapid magnetic stimulator (Magstim Company) connected with a figure-of-eight coil with a 70-mm diameter was used to deliver continuous Theta Burst stimulation. Three-pulse bursts at 50 Hz repeated every 200 ms were delivered at 80% of the active motor threshold (AMT; mean value: 34 (±5.5) % of the maximal stimulator output) for 20 sec over right BA, left BA or Cz (300 pulses). The inhibitory effect of cTBS with these characteristics last more than 15 min. [21]. AMT was tested over the contralateral motor cortex of the corresponding hemisphere for right and left BA10 sites, respectively. Electromyographic traces were recorded from the first dorsal interosseous muscle of the contralateral hand using 9-mm diameter Ag–AgCl surface cup electrodes. The active electrode was placed over the muscle belly and the reference electrode over the metacarpophalangeal joint of the index finger. Responses were amplified with a Digitimer D360 amplifier (Digitimer Ltd) through filters set at 20 Hz and 2 kHz with a sampling rate of 5 kHz and then were recorded by a computer using SIGNAL software (Cambridge Electronic Devices). We adopted a neuronavigation system (Softaxic; EMS) to position the coil precisely over the stimulation sites, using individual anatomical magnetic resonance images (MRI) at 3.0T (Siemens Magnetom Allegra; Siemens medical solution) [23]. The individual coordinates of each stimulation site were normalized a posteriori into the Montreal Neurological Institute (MNI) coordinate system and averaged. Subjects were administered the PM task in three different experimental conditions: after cTBS over right BA10 (MNI coordinates: x = 30.3±3.6; y = 50.9±3.8; z = 13.6±2.7 corresponding to the anterior portion of the right middle frontal gyrus); after cTBS over left BA10 (MNI coordinates: x = –30.3±3.4; y = 50.9±2.8; z = 13.6±1.9 corresponding to the anterior portion of the left middle frontal gyrus; Simons et al. 2006); after cTBS over a control site (MNI coordinates: x = 0±3.8; y = 0±2.7; z = 94±4.1 corresponding to the Cz position of the 10/20 EEG [electroencephalography system]). The Cz site is the most widely used control site for TMS studies because the auditory and somatosensory activations caused by vertex TMS can be equivalent to those of real TMS [24]. cTBS was delivered off-line over the target regions. After a training phase, in which subjects were instructed about the cTBS protocol and trained to perform the experimental procedure, they were administered cTBS over the target site and then performed the experimental task. The experimental procedure took less than 15 minutes to be performed. The cTBS conditions were performed in three different sessions, with an intersession interval of at least two weeks. The order of the conditions was randomized across subjects. The procedure was well tolerated by all subjects.

### PM Task

To perform the task, subjects were seated about 50 cm away from the computer screen. The experimental material consisted of a grid of 24 empty 4.5 degrees squares arranged in 4 lines of 6 columns that covered the entire screen. PM targets were 4 squares of the grid, both in central and in border positions, defined to participants before each block. Target positions were counterbalanced both within experimental conditions and between subjects. The ongoing task consisted of a sequence of 40 trials. Each trial consisted of a sequence of 4 different black squares. Each square remained black for 1.5 sec (for a total of 6 sec for each trial). Immediately after the end of the sequence, an empty grid appeared for 3 sec followed by a fixation cross that lasted 500 msec. Subjects reproduced the sequence by clicking the mouse on the correspondent square on the screen. In one block, the subject reproduced the sequence in the observed order (forward) and in another in the reverse order (backward). The PM task was to press the ‘m’ key on the keyboard whenever a target square was filled in black within each ongoing sequence. Each target position turned black twice during the block, thus PM cues appeared in 20% of the trials (n = 8). During the task, subjects had to keep the forefinger of their dominant hand on the ‘m’ key and were allowed to leave it only at the end of each trial to use the mouse to reproduce the observed sequence. Note that the ongoing task had to be performed also in the trials in which a target square appeared.

### Experimental Procedure

**Figure 2:**
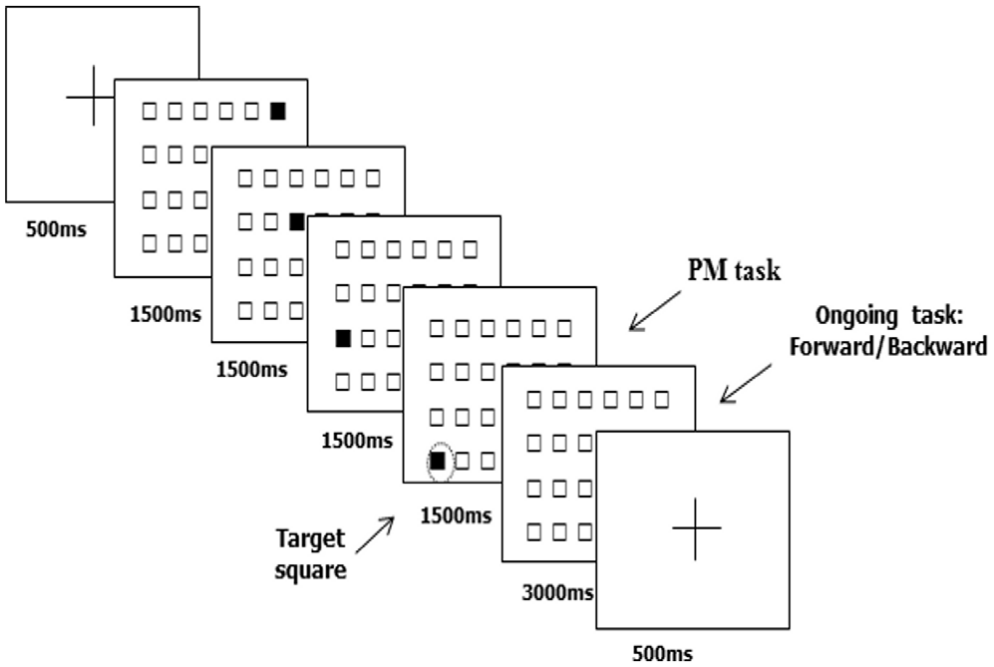
Schematic illustration of the experimental prospective memory procedure. After a study phase in which target squares were presented (here not illustrated), the subject was administered the experimental tasks in which an ongoing spatial working memory task and a PM task had to be performed. Each trial consisted of a sequence of 4 different black squares each remaining black for 1500 ms. At the occurrence of a target square subject had to press the “m” key on the keyboard (PM task). Moreover, immediately after the end of each squares sequence an empty grid appeared for 3000 ms in which subject had to reproduce the sequence in the observed or reversed order by marking the correspondent square on the screen by using the mouse (ongoing task). After a fixation cross lasting on the screen 500 ms the next trial was presented in the sequence.

The experimental procedure is schematically illustrated in Figure 2. During the experimental procedure, subjects first performed a training phase for the ongoing and PM task and then cTBS was delivered over the target site. After cTBS, subjects performed two blocks of the PM task, one forward and one backward in counterbalanced order across subjects and stimulation condition. Each block was preceded by the PM instruction to learn 4 different target positions shown on the screen. The participants were asked to reproduce them immediately on a blank grid and after a delay of about 1 min. After each PM block, a free recall and a recognition test were administered to assess memory for the target positions. The order of the site of stimulation (i.e., cTBS over right BA10, cTBS over left BA10, and cTBS over Cz) was counterbalanced across subjects. Moreover a different pair of sequences of trials and PM cue instructions, one for the forward and one for the backward condition, were used during each subject session and were counterbalanced across subjects.

To evaluate PM performance, the number of times the subject pressed the key at the occurrence of the target square (hits), response times for correct answers and number of times the subject pressed the key at the occurrence of a non-target square (false alarm) were recorded. At the end of each block, memory for target positions was assessed with two tests: free recall and, immediately after, recognition. In the former, the subject had to indicate the target positions on the screen and correct hits were recorded. In the latter, the 4 target squares and 8 filler squares were consecutively presented on the computer screen. The subject had to press a key on the keyboard when a target position was presented. An accuracy score was computed as the sum of hits and correct rejections. As for the ongoing task performance, accuracy (i.e., number of correct sequences) and response times were recorded.

## Results

Subjects’ accuracy and response times on the PM task, the ongoing tasks and the recall and recognition tests were evaluated by means of two-way ANOVAs for repeated measures with Stimulation Site (left BA 10 vs. right BA 10 vs. CZ) and Ongoing task (forward vs. backward) as within factors. To avoid the risk of alpha inflation, the p level for planned comparisons was set at 0.017, according to Bonferroni’s adjustment for multiple comparisons (i.e., 0.05/3).

### PM Task

**Figure 3:**
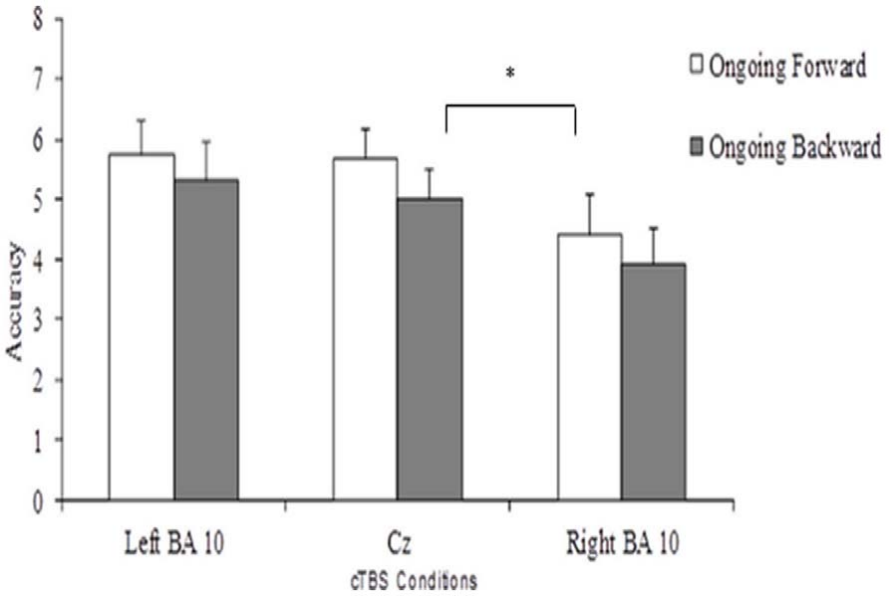
Subjects’ average accuracy on the PM task. Number of correct key presses at the occurrence of the target positions in 8 PM trials following cTBS over left frontopolar cortex (left BA10 condition), right frontopolar cortex (right BA10 condition), and Cz (Cz condition) are illustrated. Vertical lines represent standard errors. Asterisk indicates a significant difference with p<0.01 between left BA10 and the Cz condition.

Average accuracy in the three different conditions of the PM task is reported in Figure 3. The effect of Condition was significant (F(2,22) = 4.97; p = 0.016), but the Ongoing task effect (F(1,11) = 1.49; p>0.20) and the Condition*Ongoing task interaction (F(2,22) = 0.04; p>0.90) were not. Subjects were significantly less accurate after cTBS over right BA 10 (Mean = 4.17, SD = 2.2) than after cTBS over CZ (Mean = 5.33, SD = 1.7; F(1,11) = 13.1; p = 0.004; Cohen’s *d* = 0.59; Cohen 1988 [25]). They also tended to be less accurate after cTBS over right than left BA 10 (Mean = 5.54, SD = 1.9; F(1,11) = 6.15; p = 0.030; Cohen’s *d* = 0.66). No significant difference was found between left BA10 and CZ conditions (F(1,11) = 0.17; Cohen’s *d* = 0.11). In fact, passing from CZ to right BA 10 condition, 10 out of 12 subjects (about 83%) showed performance decrease with an average worsening of 3.0 (SD = 1.7), one subject improved and one exhibited unchanged performance. Conversely, compared to CZ condition, cTBS over left BA10 worsened or improved performance in a comparable number of individuals (n = 5 in both cases; about 42%) and leaved it unchanged in the remaining two subjects. We noticed two different patterns of performance. The majority of participants (n = 6) worsened PM performance only after right stimulation. Whereas, four individuals worsened their performance after both right and left stimulation with greater effect after right than left cTBS (mean = 1.9 and 1.2, respectively).

**Figure 4:**
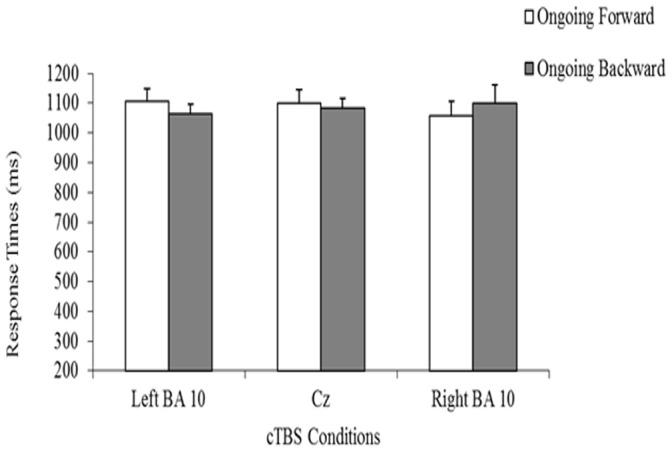
Subjects’ average response times on the PM task. Response times following cTBS over the left frontopolar cortex (left BA10 condition), right frontopolar cortex (right BA10 condition), and Cz (Cz condition) are shown. Vertical lines represent standard errors.

Response times for correct answers (Figure 4) were not affected by cTBS delivery, as documented by the absence of both the Condition F(2,22) = 0.09; p>0.90) and the Condition* Ongoing task interaction (F(2,22) = 1.81; p>0.10) effect. The Ongoing Task effect also failed to reach statistical significance (F(1.11) = 0.04; p>0.10), indicating that subjects’ response times were comparable in right BA10 (Mean = 1079, SD = 189), Cz (Mean = 1092, SD = 133) and left BA10 (Mean = 1085, SD = 128) conditions in both the ongoing forward and backward blocks.

The ANOVA applied to false alarms revealed no significant effects (Stimulation Site: F(2,22) = 0.46; p>0.60; Ongoing Task: F(1,11) = 0.05; p>0.80; Interaction: F(2,22) = 1.04; p>0.30), indicating that the number of false alarms was comparable in right BA10 (mean = 2.8, SD = 2.9), left BA 10 (mean = 2.1, SD = 2.1) and Cz (mean = 2.4, SD = 2.1) conditions and that it was not affected by the modality of square sequence repetition in the ongoing task (forward or backward).

### Ongoing Task

**Figure 5:**
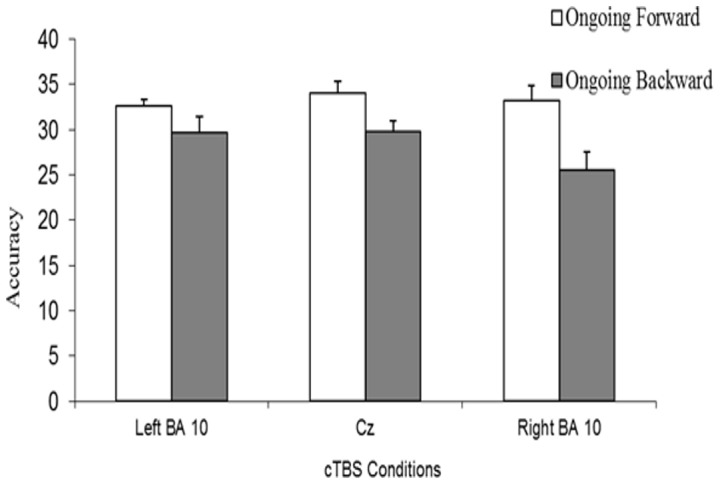
Subjects’ average accuracy score on the ongoing task. Subjects’ accuracy on 40 trials of the ongoing task after the application of cTBS over left BA10 (left BA10 condition), right BA10 (right BA10 condition), and Cz (Cz condition) is illustrated. Vertical lines represent standard errors.

Accuracy scores for the ongoing tasks are presented in Figure 5. A two-way ANOVA revealed a significant effect of the Ongoing Task (F(1,11) = 20.9; p<0.001; Cohen’s *d* = 0.94); indeed, across the three experimental conditions, subjects were more accurate on the forward than the backward task (Mean = 33.3, SD = 4.3 and Mean = 28.3, SD = 6.13, respectively). However, no significant effect of the Stimulation site and Stimulation Site*Ongoing Task interaction was found (F(2,22) = 2.09 and F(2,22) = 2.55, respectively; p>0.10 in both cases). This indicates that the subjects were comparably accurate on the ongoing task after cTBS over right BA 10 (Mean = 29.37, SD = 7.4), left BA 10 (Mean = 31.1, SD = 4.9) and CZ (Mean = 31.9, SD = 4.8) regardless of whether the spatial sequences had to be reproduced forward or backward.

### Recollection of Target Positions

#### Free-recall test

The two-way ANOVA revealed no significant effect of the two main factors, that is, Condition (F(2,22) = 0.15; p>0.80) and Ongoing Task (F(1,11) = 3.14; p>0.10) or of the Condition* Ongoing Task interaction (F(2,22) = 0.15; p>0.80). This indicates that the mean number of target positions correctly recalled was comparable following cTBS over right BA 10 (Mean = 3.9; SD = 0.2), left BA 10 (Mean = 3.9, SD = 0.2) and CZ (Mean = 3.9, SD = 0.4) regardless of the difficulty of the ongoing task.

#### Recognition test

Also in this case, the absence of significant Condition (F(2,22) = 0.29; p>0.70), Ongoing Task (F(1,11) = 0.19; p>0.60) and Condition* Ongoing Task interaction (F(2,22) = 0.01; p>0.90) effects documents comparable target recognition accuracy following cTBS over right BA 10 (Mean = 11.3; SD = 0.3), left BA 10 (Mean = 11.0, SD = 0.3) and CZ (Mean = 11.0, SD = 0.5 ).

## Discussion

The first finding of the study is confirmation that the frontopolar cortex is involved in the mediation of PM processes. In fact, the subjects who participated in the experiment were significantly less accurate on the PM task following inhibitory cTBS over right BA 10 than after cTBS over a control site. These findings are quite consistent with those of previous functional neuroimaging investigations that demonstrated the involvement of BA 10 in the neural control of PM processes [2], [4]–[7]; for a review, see [3]). Moreover, the effect of cTBS over right BA 10 on PM performance was quite specific. Indeed, subjects’ episodic recollection of the PM targets (as expressed by performance accuracy on both recall and recognition tests), accuracy on the visual-spatial working memory ongoing task and rate of false alarms were not affected by cTBS over right BA10. Moreover, the detrimental effect of cTBS over right BA 10 on PM accuracy was not modulated by the difficulty of the ongoing task. These findings are congruent with previous results indicating a specific association between BA 10 activity and PM operations that is not mediated by episodic memory, executive functioning or working memory processes [8], [9].

The main finding of this study is that inhibition of right but not left lateral BA 10 significantly worsened subjects’ accuracy on an event-based visual-spatial PM paradigm. This result is specular to that of a previous study in which we documented a detrimental effect of inhibitory cTBS over left but not right BA 10 on the PM scores of a similar sample of healthy subjects [9]. The technical characteristics of the cTBS procedure as well as the MNI coordinates for brain site stimulation were exactly the same in the two studies. The procedural features of the PM task were also the same in the two studies, except that here they consisted of visual-spatial stimuli and in the previous study they consisted of written words for both the PM targets and the ongoing task. Therefore, it seems unlikely that the differential sequelae of cTBS over right and left BA 10 on PM performance observed in the two studies is due to factors other than the physical characteristics of the experimental material. It should be noted, however, that the two PM procedures do not seem fully comparable as to level of difficulty. In fact, on average the subjects in the present study had poorer total PM accuracy (about 63%) and higher response latencies (1085 ms) than those who participated in the previous experiment (accuracy = about 68%; response time = 816 ms).

Taken together, the previous data providing evidence of left BA10 involvement in verbal PM and the present findings of right BA 10 involvement in visual-spatial PM suggest the presence of a material-specific lateralization of PM processes in BA 10. As previously discussed, this issue has been poorly explored and extant data are not univocal. Indeed, changes in left frontopolar cortex hemodynamic responses while subjects performed PM tasks were found regardless of the kind of material used (e.g., shapes, letters; [5], [13]). According to these and other concurrent findings on the preferential involvement of the left rostral prefrontal cortex in PM and future imagery [12], a specialized role for this region in general prospective thinking (i.e., prospection) was hypothesized. However, some findings indicate the presence of a differential involvement of left and right BA 10 depending on the cognitive demand of the PM task [14] and the phases of the PM process the subject is engaged in [13]. Moreover, other authors failed to find any differential recruitment of the two hemispheres at the level of the frontal pole [4], [7]. For the purpose of our discussion, it should be noted that functional neuroimaging paradigms were used in all of the above-mentioned studies. Indeed, application of TMS allows investigating the effect of a transient interference of the activity of a circumscribed cortical region on a certain mental function, whereas functional neuroimaging allows observing the occurrence of concomitant phenomenon (i.e., behavioural and brain responses). Therefore, it is difficult to directly compare results deriving from the application of these two methods, particularly when they are not fully congruent. By contrast, from a conceptual perspective the TMS condition seems much more comparable to a naturalistic situation in which the subject experiences decreased cognitive functioning as a consequence of brain injury. In this regard, Uretzky and Gilboa [26] recently published a single-case study in which they reported the effect of right frontopolar damage (i.e., atrophy) due to head trauma on PM tasks involving verbal material (i.e., words). Indeed, their subject performed poorly on all PM tasks administered. The portions of BA 10 affected by brain damage in this patient appear broad enough to include the medial part; in our study, however, we stimulated a region on the scalp that corresponded to a smaller and more lateralized sub-region of the anterior prefrontal cortex. In a further study with individuals with brain lesions, Volle et al. [15] failed in demonstrate a differential association between left/right frontopolar cortex and subjects’ performance on verbal and visual (i.e., pictures representing concrete stimuli) event-based PM tasks. However, the authors found a significant relationship between the presence of lesions involving right frontopolar cortex and subjects’ performance on time-based PM procedures. Indeed, as the authors argue, time-based paradigms require subjects to perform an action after a certain time interval is elapsed implicating different cognitive processes from those involved in event-based paradigms (e.g., time estimation). However discussion of this topic is limited because, it is almost impossible to study patients with selective focal lesions of the frontopolar cortex.

The possible material-specific lateralization of PM functioning in BA 10 suggests that the organization of neuronal activity in this area of the prefrontal cortex is also modulated by the physical features of the stimuli. This hypothesis is inconsistent with the idea that involvement of BA 10 in cognitive tasks is substantially content-independent [3], [16]. According to this view, BA 10 participates in PM operations by implementing abstract processes, allowing the coordination of multiple hierarchical organized activities (branching) [18], [27], and by modulating the equilibrium between stimulus-oriented and stimulus-independent behaviour (i.e., gateway hypothesis [3], [20]). As previously stated, this view is congruent with the idea of a hierarchical rostral-caudal organization of the prefrontal cortex and BA10, in which the most anterior portions are involved in highly integrative, associative and supervisory operations [17]–[20]. In this regard, it should be noted that here, as well as in our previous cTBS study [9], we delivered cTBS over a relatively posterior region of lateral BA 10. It could be argued that the effect we found in our two studies would not have been observed if cTBS had been delivered over more rostral portions. It could be also argued that in our experimental paradigm in which the PM cues appeared with a relatively high frequency (20%), the necessity to implement abilities allowing the successful switching between different cognitive operations, abilities that are needed for successfully facing with PM tasks per se, was stressed. It should be noted that a similar frequency of occurrence of the PM target has been used in neuroimaging studies that also highlighted an involvement of BA10 [5]; [8]. At this regard, it has been proposed that the lateral portion of BA 10 is particularly involved in switching attention between ongoing task/target detection and the internal representation of intention [28]. Within this view, the hypothesis could be formulated that performance decrease observed after functional inhibition of right frontopolar cortex may be due to an attentional imbalance between these two phases. This imbalance could have affected subject’s ability to activate the plan aimed at realizing the intention [9]. To limit our discussion of this point, note that we did not manipulate factors other than ongoing task complexity, that could have allowed us to make reliable inferences about the cognitive mechanisms underlying PM decline after cTBS delivery or the exact phase of the PM process (i.e. encoding, maintenance, retrieval or execution phase) that is impaired.

The idea that activity in BA 10 can be modulated by the specific characteristics and presentation modalities of stimuli is also supported by some functional neuroimaging data that show a significant effect of content-related manipulations on frontal pole activations [13], [14], [29]. In an event-related functional magnetic resonance investigation in healthy individuals, Rea et al. [29] explored hemodynamic changes in the frontal pole as a function of the emotional valence of PM targets (pictures representing human facial expressions). Their results show a significant effect of the manipulation of the emotional features of the stimuli in the rostral prefrontal cortex; increased activity of right BA 10 was associated with the elaboration of emotional cues (with respect to neutral cues).

In conclusion, here we investigated the role of the left and right frontal poles on PM functioning. Our results provide a novel TMS finding on the role of frontal pole in prospective memory functioning. In particular, here we demonstrated that transient cTBS inhibition of right lateral BA 10 affects subjects’ performance on a visual-spatial PM task. Taking into account previous findings of a significant association between the left frontal pole and verbal PM, the present findings can be interpreted in the context of a material-specific lateralization of PM in the prefrontal cortex, thus, suggesting that activity in the rostral prefrontal areas is not completely stimulus independent.
